# Multilayer Polyethylene Separator with Enhanced Thermal and Electrochemical Performance for Lithium-Ion Batteries

**DOI:** 10.3390/ma19020342

**Published:** 2026-01-15

**Authors:** Jingju Liu, Baohui Chen, Jiarui Liu, Luojia Chen, Jiangfeng Wang, Kuo Chen, Zuosheng Li, Chuanping Wu, Xuanlin Gong, Linjin Xie, Jin Cai

**Affiliations:** 1 State Key Laboratory of Disaster Prevention & Reduction for Power Grid, State Grid Hunan Electric Company Limited Disaster Prevention and Reduction Center, Changsha 410007, China; bymountains@gmail.com (B.C.); jiaruiliu0188@163.com (J.L.); 13717955979@163.com (L.C.); wangjf@pku.edu.cn (J.W.); sy1901324@buaa.edu.cn (K.C.); i-skolf@139.com (Z.L.); jandom@126.com (C.W.); 2 Hunan Disaster Prevention Technology Co., Ltd., Changsha 410100, China; 119031910101@sjtu.edu.cn (X.G.); 18684788989@163.com (L.X.); m13875802560@163.com (J.C.)

**Keywords:** polyethylene wax microspheres, para-aramid, aluminium oxide, high safety, lithium-ion battery

## Abstract

The inherent limitations of conventional polyolefin separators, particularly their poor thermal stability and insufficient mechanical strength, pose significant safety risks for lithium-ion batteries (LIBs) by increasing susceptibility to thermal runaway. In this study, we developed a novel multilayer separator through sequential coating of a commercial polyethylene (PE) substrate with aluminum oxide (Al_2_O_3_), para-aramid (PA), and polyethylene wax microspheres (PEWMs) using a scalable micro-gravure process, denoted as SAPEAS, signifying a PE-based asymmetric structure separator with enhanced thermal shutdown and dimensional stability. The SAPEAS separator exhibits an early thermal shutdown capability at 105 °C, maintains structural integrity with negligible shrinkage at 180 °C, and demonstrates comprehensive performance enhancements, including enhanced mechanical strength (tensile strength: 212.3 MPa; puncture strength: 0.64 kgf), excellent electrolyte wettability (contact angle: 12.8°), a high Li^+^ transference number (0.71), superior ionic conductivity (0.462 mS cm^−1^), outperforming that of commercial PE separators. In practical LFP|Gr pouch cells with ampere-hour (Ah) level capacity, the SAPEAS separator enables exceptional cycling stability with 97.9% energy retention after 1000 cycles, while significantly improving overcharge tolerance compared to PE. This work provides an effective strategy for simultaneously improving the safety and electrochemical performance of LIBs.

## 1. Introduction

Lithium-ion batteries (LIBs) have become the cornerstone energy storage technology for portable electronics, electric vehicles, and large-scale stationary energy storage systems due to their high energy density, excellent cost-effectiveness, and long cycle life [1,2,3]. However, safety concerns, particularly thermal runaway incidents leading to fires, have been frequently reported in electric vehicles and grid-scale storage installations, significantly impeding broader application [4,5,6,7]. Thermal runaway typically originates from uncontrolled exothermic reactions within the battery, with separator failure serving as a key initiating factor [8,9,10]. As a critical internal component, the separator must not only facilitate efficient ion transport but also prevent direct contact between the cathode and anode to ensure operational safety. Polyolefin microporous separators, such as polyethylene (PE) and polypropylene (PP), are widely used as commercial separators in LIBs owing to their favorable mechanical properties, chemical stability, and mature manufacturing processes [11,12]. Nevertheless, their limited thermal stability often results in severe shrinkage at elevated temperatures, causing internal short circuits and potentially triggering thermal runaway or even explosion [13,14,15]. Consequently, developing advanced separators with enhanced thermal safety is crucial for improving the overall reliability and safety of LIBs [16,17].

To enhance the thermal safety of separators, researchers have proposed incorporating a “thermal shutdown” function [18,19,20,21]. This mechanism utilizes low-melting-point materials that melt at a specific temperature, sealing the separator pores to block ion transport and terminate battery operation to prevent thermal runaway occurs [22,23]. However, even after pore closure, residual heat accumulated within the battery may continue to raise the internal temperature. If the separator lacks sufficient thermal resistance, it may undergo shrinkage or melting, failing to effectively avert thermal runaway [24,25]. Some thermal shutdown separators can maintain the closed-pore state for only approximately three minutes, which is insufficient under conditions of sustained temperature elevation [26]. Therefore, concurrently lowering the shutdown temperature and improving the thermal stability of the separator presents a promising strategy to enhance its thermal safety performance [27].

Regarding the reduction in the shutdown temperature, low-melting-point polymers such as ethylene-vinyl acetate (EVA) copolymer have been investigated as functional coatings [28,29]. However, these materials often exhibit poor compatibility with electrolytes, leading to swelling or dissolution, which adversely affects battery performance. Moreover, commercial cell production involves a drying step above 80 °C to remove moisture. If the separator’s shutdown temperature falls below this threshold, pore sealing occurs during normal manufacturing, severely compromising process feasibility [30]. Therefore, developing functional separators that combine an appropriate shutdown temperature, good electrolyte compatibility, and high thermal stability remains a significant challenge.

To enhance the thermal stability of shutdown separators, several studies have focused on incorporating high-temperature-resistant scaffold materials [31,32,33,34,35,36,37]. For instance, Kim et al. constructed a polyester@polyethylene (PES@PE) composite separator via dip-coating, achieving shutdown at 140 °C while maintaining structural integrity up to 200 °C [31]. Wei et al. fabricated a core–shell separator of polyacrylonitrile@poly(butylene succinate) (PAN@PBS) using coaxial electrospinning, which exhibited a rapid shutdown response at 110 °C and retained structural stability up to 250 °C, integrating high thermal sensitivity and exceptional thermal stability [34]. Lu et al. developed a sandwich-structured nanofiber composite separator (denoted as PPPA) composed of PAN, PBS, polyethylene glycol (PEG), and ammonium polyphosphate (APP) through coaxial electrospinning, spraying, and calendering techniques, endowing it with comprehensive thermal protection capabilities, including thermal energy storage, circuit-breaker function, dimensional stability, and flame retardancy [37]. Although these approaches have improved the thermal safety of separators to some extent, their fabrication mostly relies on complex synthesis procedures or inefficient processing methods, and often fails to address the synergistic optimization of other key application properties such as wettability, porosity, and mechanical strength, thereby limiting their practical application potential.

From an industrial perspective, the thermal shutdown separator remains highly attractive for lithium-ion battery safety due to reliability, cost-effectiveness, and ease of integration. To address the aforementioned challenges, this work develops a multilayer separator by sequentially coating a PE substrate with rigid aluminum oxide (Al_2_O_3_) particles, a high-temperature-resistant para-aramid (PA) with continuous network structure, and thermally responsive polyethylene wax microspheres (PEWMs). The resulting separator is designated as SAPEAS, signifying a PE-based asymmetric structure separator with enhanced thermal shutdown and dimensional stability. Compared with commercial PE separators, the fabricated SAPEAS separator exhibits an efficient thermal shutdown capability at 105 °C, along with enhanced mechanical strength, superior thermal stability, improved electrolyte wettability, higher Li^+^ transference number, and increased ionic conductivity. When implemented in practical pouch-type LFP|Gr cells with ampere-hour (Ah) level capacity, the SAPEAS separator delivers exceptional performance, achieving 97.9% capacity retention after 1000 cycles and exhibiting improved overcharge tolerance compared to PE separators. These results validate that the SAPEAS separator possesses both remarkable thermal safety and superior electrochemical performance. This work presents a functional and readily scalable approach for advancing next-generation battery separators.

## 2. Materials and Methods

### 2.1. Materials

No further purification was applied to the commercially sourced reagents prior to use. Materials used for fabricating the SAPEAS separator, including polyethylene (PE) separator with a thickness of 9 μm (procured from Shenzhen Xuran Electronic Co., Ltd., Shenzhen, China), aluminum oxide (Al_2_O_3_) powder with a D_50_ of 0.8 μm (purchased from Anhui Yishi New Material Technology Co., Ltd., Jinhua, China), Poly(p-phenylene terephthalamide) (para-aramid, PA) solution (supplied by Yantai Xinghe Battery Materials Technology Co., Ltd., Yantai, China), and polyethylene wax microspheres (PEWMs) solution with a particle size D_50_ of 0.7 μm (obtained from Nanjing Tianshi Advanced Materials Co., Ltd., Jinzhou, China), were all of commercial grade. Additives for the coating slurry preparation, namely acrylic polymer and disodium succinate, were supplied by Shanghai Yingcheng Chemical Co., Ltd. (Shanghai, China), and modified polyacrylate was provided by Shenzhen Yanyi New Material Co., Ltd. (Shenzhen, China) For electrode preparation, lithium iron phosphate (LiFePO_4_, LFP) and graphite (Gr) were provided by Hunan Yuneng New Energy Battery Material Co., Ltd. (Xiangtan, China) and Hunan Rongli New Energy Technology Co., Ltd. (Changsha, China), respectively. The electrolyte was supplied by Hunan Faraight New Energy Technology Co., Ltd. (Changsha, China). The coin cell components, including lithium foils, shrapnel and stainless steel spacers, were sourced from Shenzhen Kejingzhida Technology Co., Ltd. (Shenzhen, China).

### 2.2. Preparation of Separator

**Scheme A1:**
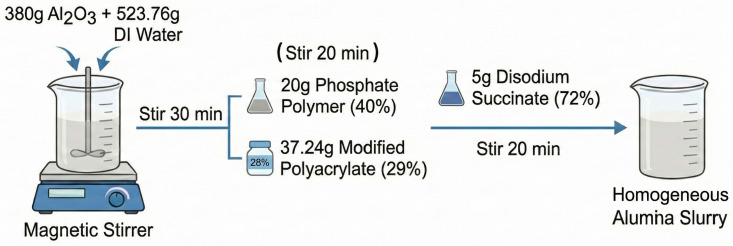
Schematic illustration of the preparation process of the Al_2_O_3_ slurry.

**Scheme A2:**
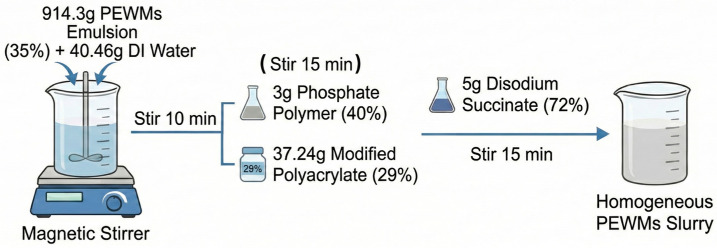
Schematic illustration of the preparation process of the PEWMs slurry.

The Al_2_O_3_ coating slurry was prepared by first dispersing 380 g of Al_2_O_3_ in 523.76 g of deionized water (Tangshi Kangning Technology Development Co., Ltd., Chengdu, China) under continuous stirring for 30 min. Subsequently, 20 g of a phosphate polymer (40 wt% solid content, purchased from Shanghai Yingcheng Chemical Co., Ltd., Shanghai, China) and 37.24 g of modified polyacrylate (29 wt% solid content) were added sequentially, each followed by 20 min of stirring. Finally, 5 g of disodium succinate (72 wt% solid content) was introduced, and the mixture was stirred to form a homogeneous Al_2_O_3_ slurry. The fabrication process of the Al_2_O_3_ slurry is illustrated in Scheme A1. A similar procedure was employed to prepare a 32 wt% PEWMs thermosensitive coating slurry using the same auxiliary agents. The fabrication process of the PEWMs slurry is illustrated in Scheme A2.

**Scheme 1:**
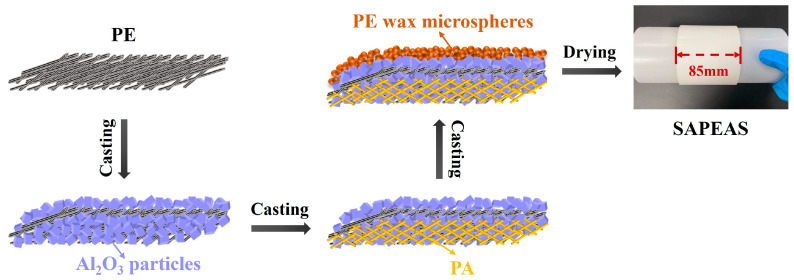
Schematic illustration for preparation process of SAPEAS separators.

Using a micro-gravure coater (Jiatuo Intelligent Equipment Co., Ltd., Dongguan, China), as shown in Scheme 1, the prepared Al_2_O_3_ slurry was first applied onto both sides (A and B) of the PE separator, with the dry coating thickness controlled at 1.5 μm per side. Thereafter, the PA solution and the thermal-sensitive slurry were coated onto side A and side B, respectively, each also achieving a dry thickness of 2 μm. The resulting multilayer composite separator, with a total width of 85 mm and a total coating thickness of 7 μm, was designated as SAPEAS.

### 2.3. Preparation of LFP|Gr Pouch Cell

To evaluate the electrochemical and safety performance of the separators, an LFP|Gr pouch cell with a nominal capacity of 1.6 Ah was assembled in a controlled environment with low humidity and particulate levels. The cell was constructed by stacking 10 pieces of LFP electrodes (39 mm × 79 mm), 11 pieces of Gr electrodes (39 mm × 80.5 mm), and approximately 3 m of separator (85 mm width, either SAPEAS or PE), with an electrolyte dosage of 4 g Ah^−1^. Prior to electrolyte injection, the pouch cells were vacuum-dried at 90 °C for 24 h. During the formation process, all pouch cells were charged at 0.1C for 10 min, followed by 0.16C for 110 min at 45 °C under an external pressure of 700 kPa. The gas pouch was then removed, and the cells were finally vacuum-sealed. These pouch cells were initially activated on a LAND battery test system (Wuhan Land Electronics Co., Ltd., Wuhan, China) by cycling at 0.1C within a voltage range of 2.5 V to 3.65 V.

### 2.4. Electrochemical Measurement

The temperature-dependent ionic resistance of the separator was determined from the alternating-current (AC) impedance of stainless steel (SS)|separator|SS coin cells measured at various temperatures. The impedance spectra were acquired at 100 kHz with an amplitude of 5 mV using a CHI660e electrochemical workstation (Chenhua Instruments, Shanghai, China). Temperature control during the measurements was maintained using a forced-air drying oven (Hefei Kejing Material Technology Co., Ltd., Hefei, China).

The electrochemical stability of the separators was evaluated by linear sweep voltammetry (LSV) using an Li|separator|SS cell, with the potential scanned between the open-circuit potential (OCP) and 5.8 V (vs. Li^+^/Li) at a rate of 5 mV s^−1^.

The ionic conductivity (*σ*) of the separators in the electrolyte-soaked state was determined through AC impedance spectroscopy. The measurement was conducted on a CHI660e electrochemical workstation using symmetric SS|separator|SS coin cells, with an applied frequency sweeping from 10^5^ to 10^−1^ Hz. The value of *σ* was subsequently calculated using Equation (1):

(1) $$\sigma =\frac{d}{R_{b}\times S}$$

where *d* is the separator thickness, *R_b_* is the bulk resistance, and *S* represents the effective area.

The Li^+^ transference number (*t*_Li_^+^) of the separators was determined by combining direct current (DC) polarization and AC impedance measurements in a symmetric Li|separator|Li cell. The protocol involved applying a constant DC bias of 10 mV (ΔV) and monitoring the current until it stabilized. The interfacial resistance was recorded via impedance spectroscopy both before and after the polarization step. The value of *t*_Li_^+^ was then calculated using Equation (2):

(2) $$t_{{\mathrm{Li}}^{+}}=\frac{I_{s}\left(\Delta V-I_{0}R_{0}\right)}{I_{0}\left(\Delta V-I_{s}R_{s}\right)}$$

where *I*_0_ and *I_s_* are the initial and steady-state currents, respectively, and *R*_0_ and *R*_s_ represent the corresponding interfacial resistances at these stages.

The long-term cycling stability and rate capability tests of the pouch cells were carried out using a LAND battery test system (Wuhan Land Electronics Co., Ltd., Wuhan, China) within a voltage window of 2.5–3.65 V. It should be noted that, to ensure a direct and equitable comparison, the long-term cycling tests for both types of separator batteries were conducted under identical laboratory conditions using adjacent channels of the same multi-channel (8-channel) battery charge–discharge system.

### 2.5. High-Temperature Overcharge Test

The high-temperature overcharge test was conducted according to the following procedure: First, the state of charge (SOC) of the battery was adjusted to 100%. The fully charged battery was then continuously charged at a 2C rate under an ambient temperature of 80 °C, while its surface temperature was monitored simultaneously. The test was terminated when the battery voltage reached 19.2 V, which corresponds to six times the nominal voltage of a single cell (3.2 V).

### 2.6. Characterization

The surface morphology was characterized by scanning electron microscopy (SEM, SU8010, Hitachi, Japan) with energy dispersive X-ray spectroscopy (EDS). X-ray diffraction (XRD) analysis was conducted on a Rigaku Ultima IV system (Rigaku Corporation, Tokyo, Japan). The tensile strength and puncture strength of the separators were measured using an INSTRON 3366 universal testing machine (Instron Corporation, Norwood, MA, USA). The temperature-dependent deformation was evaluated using a thermomechanical analyzer (TMA/SDTA 2+, Mettler Toledo, Greifensee, Switzerland) by monitoring the sample dimension variation under a controlled temperature program. Simultaneous differential scanning calorimetry (DSC) and thermogravimetric (TG) analyses were performed using a STA 449 F3 Jupiter TG-DSC synchronous thermal analyzer (NETZSCH, Selb, Germany) under a nitrogen atmosphere at 10 °C/min. Contact angle measurements were performed on a Data Physics OCA20 instrument (DataPhysics Instruments GmbH, Filderstadt, Germany) at room temperature with 3 μL droplets of the EC:DMC-based electrolyte containing 1 M LiPF_6_. Each reported data point is the average of three measurements obtained from distinct positions on the membrane surface. Separators acquired from the battery after overcharge were thoroughly washed with dimethyl carbonate (DMC, Guangzhou Tinci Materials Technology Co., Ltd., Guangzhou, China) and dried in an Ar-filled glovebox prior to transfer for SEM analysis.

## 3. Results

The SAPEAS separator is fabricated by sequentially coating both sides of a polyethylene (PE) separator with thin layers of rigid aluminum oxide (Al_2_O_3_) particles, a high-temperature-resistant para-aramid (PA) with a continuous network structure, and thermally responsive polyethylene wax microspheres (PEWMs), as illustrated in Scheme 1. The preparation employs a widely used micro-gravure casting process, which offers ease of implementation.

**Figure 1:**
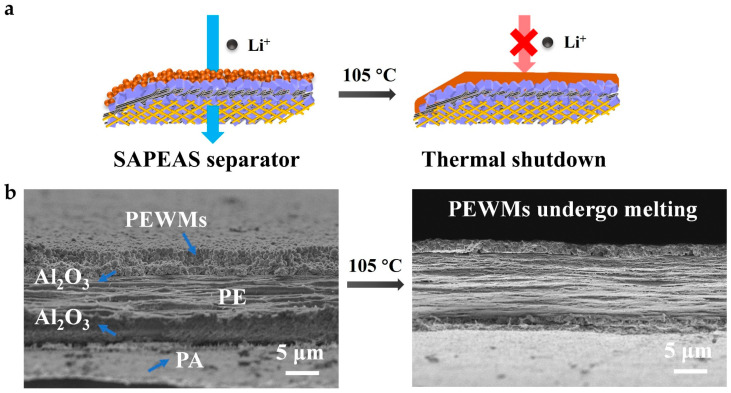
(**a**) Schematic illustration of the thermal shutdown process of the separator. (**b**) Cross-sectional SEM images of the separators before and after the thermal shutdown process.

At room temperature, the SAPEAS separator functions normally. The coating composed of PEWMs retains a porous structure (Figure 1a,b, left), which enables free Li^+^ transport and thereby guarantees the normal operation of the battery. When the temperature reaches the melting point of the PEWMs (approximately 105 °C), these microspheres melt, collapse, and coalesce. This phase transition results in the formation of a dense and continuous barrier layer from the previously porous coating, as shown in Figure 1a,b (right). Consequently, the ion-conducting pores within the coating are sealed, effectively blocking the Li^+^ conduction path between the electrodes and rapidly terminating the electrochemical reaction. This thermal shutdown process, directly facilitated by the phase change in the PEWMs, is of great significance for preventing the occurrence of thermal runaway reactions under abusive conditions. Furthermore, owing to the thermally resistant Al_2_O_3_ and PA modified layers, the SAPEAS separator exhibits remarkable thermal stability up to 180 °C, effectively preventing short circuits under high-temperature conditions.

### 3.1. Composition and Morphology Characterization

**Figure 2:**
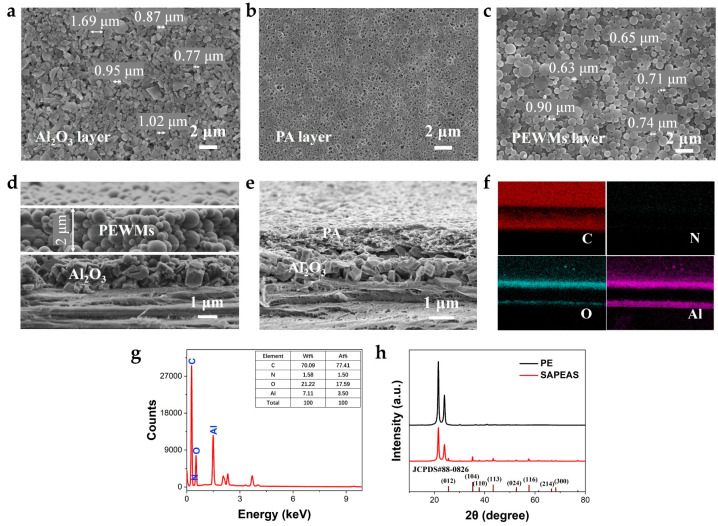
Composition and morphological characterization of the SAPEAS separator. (**a**–**e**) Surface and cross-sectional SEM images of SAPEAS. (**f**) Elemental mapping and (**g**) corresponding element distribution analysis across the cross-section of SAPEAS. (**h**) XRD patterns of the PE and SAPEAS separators.

The pore structure and surface morphology of the separator are critical for preventing direct contact between the cathode and anode while facilitating efficient lithium-ion (Li^+^) transport [38]. Figure 2a–c shows scanning electron microscopy (SEM) images of the Al_2_O_3_, PA, and PEWMs layers coated on the SAPEAS separator. These images demonstrate that the Al_2_O_3_ particles (Figure 2a), the PA with a continuous network structure (Figure 2b), and the PEWMs (Figure 2c) are uniformly distributed without evident agglomeration. Cross-sectional SEM images clearly reveal the hierarchical architecture of the SAPEAS separator (Figure 1b, left). The Al_2_O_3_ particles are densely and uniformly arranged on both sides of the PE substrate. On one side of the Al_2_O_3_ layer, the PEWMs are homogeneously distributed (Figure 2d), while the opposite side is coated with a porous polyamide (PA) network featuring well-defined and uniformly sized nanopores (Figure 2e). Cross-sectional energy dispersive X-ray spectroscopy (EDS) confirms the uniform distribution of C, N, O, and Al elements, as well as their relative content ratios (Figure 2f,g), providing definitive evidence for the successful coating of functional layers on the PE substrate. The presence of abundant oxygen species and highly tortuous nanopores in the Al_2_O_3_ and PA layers promotes superior electrolyte wettability and uptake [38,39], thereby improving the overall electrochemical performance of the LIBs.

The crystal structure of the SAPEAS separator was characterized by X-ray diffraction (XRD). As shown in Figure 2h, all separator samples display two distinct diffraction peaks at 21.6° and 24.0°, corresponding to the (110) and (200) crystallographic planes of PE, respectively [27]. For the SAPEAS separator, several additional diffraction peaks are observed and can be assigned to the crystal planes of Al_2_O_3_ (JCPDS #88-0826). These results confirm the successful fabrication of the multilayered SAPEAS separator.

### 3.2. Thermal Shutdown Functionality

**Figure 3:**
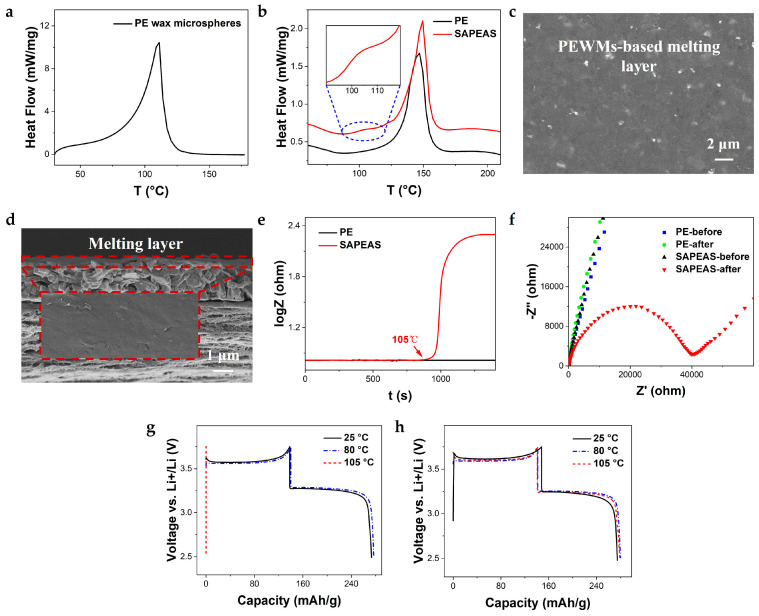
Thermal shutdown function evaluation of SAPEAS. (**a**,**b**) DSC curves of (**a**) PE wax microspheres and (**b**) the pristine PE versus SAPEAS separators. (**c**,**d**) Surface and cross-section SEM images of SAPEAS after heat treatment at 105 °C. (**e**) Log Z–t plots of the SS/separator/SS coin cells using PE and SAPEAS separators during heating, (**f**) Corresponding EIS plots of the same cells before and after heating. (**g**,**h**) Charge/discharge curves of the LiFePO_4_/separator/Li coin cells using (**g**) SAPEAS and (**h**) PE separators at various temperatures ranging from 25 °C to 105 °C.

Melting temperature and melting rate are two critical factors that determine the protective performance of thermal-shutdown separators. In principle, an effective thermal-shutdown layer should have a melting point slightly above the maximum allowable operating temperature of LIBs (∼80 °C) but below that of the separator substrate, ensuring dimensional stability during the thermal-shutdown process [30,40]. The melting behaviors of PEWMs, SAPEAS separator, and PE separator were analyzed using differential scanning calorimetry (DSC). As shown in Figure 3a, a broad endothermic transition with a peak temperature (*T_m_*) of approximately 105 °C is observed, which corresponds to the melting of PEWMs. This *T_m_* is nearly ideal for triggering thermal shutdown, as it allows the microporous PE substrate to maintain structural integrity. Moreover, this melting point ensures that the separator remains thermally stable during battery manufacturing and testing processes involving elevated temperatures [6,9,10]. In the DSC curve of the SAPEAS separator (Figure 3b), an endothermic peak at 105 °C is also present, which can be attributed to the melting of the PEWMs coating. These results demonstrate that the SAPEAS separator incorporating PEWMs exhibits strong potential for reliable thermal shutdown functionality at around 105 °C.

The thermal shutdown behavior of PEWMs can be elucidated by analyzing the morphological evolution of the PEWMs-coated separator at different temperatures. As shown in Figure 2c,d, the randomly stacked microspheres on the Al_2_O_3_-coated surface form a porous structure that ensures ample pathways for Li^+^ transport. Upon reaching 105 °C, the PEWMs begin to melt and then fuse together, effectively blocking off the pores within the coating layer (Figure 3c,d). This effectively disrupts Li^+^ conduction between the electrodes, thereby terminating the battery reaction and preventing thermal runaway.

The thermal-response characteristics of the thermal-shutdown separator were further evaluated by monitoring the evolution of AC impedance during heating. Figure 3e shows the impedance variation in PE and SAPEAS separators over a temperature range of 25 °C to 105 °C. For the SAPEAS separator, the impedance began to increase rapidly upon reaching 105 °C and then plateaued, indicating a fast and effective thermal-shutdown response. This behavior is further confirmed by the corresponding impedance spectra before and after heating at 105 °C (Figure 3f). In contrast, the impedance of the PE separator remained largely unchanged, as the temperature was still below its closure temperature (~135 °C) [21]. This earlier shutdown and fast thermal response of the SAPEAS separator offer enhanced and reliable safety protection against thermal runaway in LIBs.

The thermal-shutdown behavior of the SAPEAS separator was further validated by evaluating the charge–discharge performance of LiFePO_4_/Li coin cells at elevated temperatures. As shown in Figure 3g,h, the cell with the SAPEAS separator exhibited highly reversible charge–discharge behavior and delivered a discharge capacity of approximately 145 mAh g^−1^ across the temperature range of 25 °C to 80 °C, outperforming the cell with PE. However, when the temperature reached 105 °C, the cell almost ceased to charge or discharge; the charge voltage rapidly increased to the upper limit of 3.65 V, while the discharge voltage sharply dropped to the lower limit of 2.5 V, resulting in no detectable capacity. This indicates the activation of the thermal-shutdown function. In contrast, the cell with a conventional PE separator remained operating at 105 °C, posing potential safety hazards [21]. These findings clearly demonstrate that, unlike the PE separator, the SAPEAS separator can effectively trigger thermal shutdown at 105 °C, thereby reliably improving the safety of LIBs.

### 3.3. Thermal Stability and Mechanical Strength

In LIBs, separators exhibiting poor thermal stability and insufficient mechanical strength present significant safety hazards, as they may cause internal short circuits, leading to thermal runaway or even explosions [27]. To ensure operational safety, separators must possess high thermal stability and robust mechanical properties to endure extreme operating conditions [36]. A systematic evaluation was thus conducted, encompassing flame retardancy, dimensional stability, and thermal decomposition tests, to comprehensively assess the safety performance.

**Figure 4:**
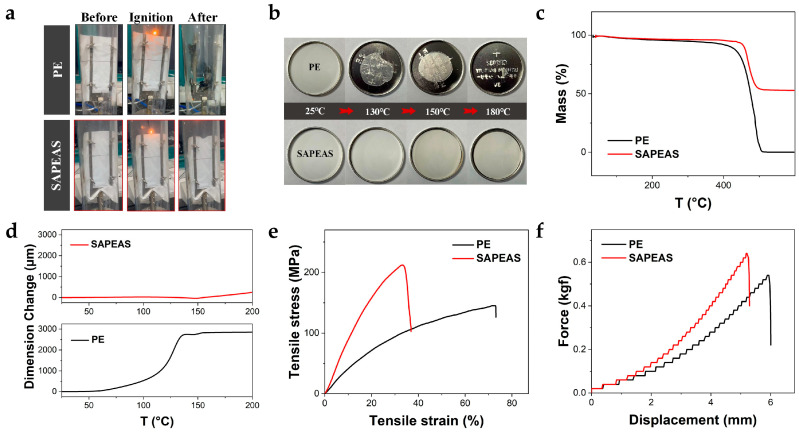
Thermal stability and mechanical property evaluation of SAPEAS. (**a**) Fire-resistant tests of PE and SAPEAS separators. (**b**) Thermal shrinkage images of PE and SAPEAS separators. (**c**) TGA curves of PE and SAPEAS. (**d**) Deformation-temperature curves of PE and SAPEAS. (**e**) Tensile stress–strain curves of PE and SAPEAS. (**f**) Puncture force-displacement curves of PE and SAPEAS.

As shown in Figure 4a, the PE separator undergoes significant shrinkage upon direct flame exposure and is completely consumed within seconds (≤5 s), whereas the SAPEAS separator exhibits negligible shrinkage and demonstrates self-extinguishing behavior. This favorable flame-retardant performance can be attributed to the high limiting oxygen index (∼29%) inherent in the aramid-based material [38]. Furthermore, the thermal shrinkage of the SAPEAS separator was evaluated and compared with that of the PE separator by measuring dimensional changes after heat treatment (Figure 4b). When heated to 130 °C, the PE separator exhibited noticeable dimensional reduction, followed by severe shrinkage exceeding 50% at 150 °C, and nearly complete collapse at 180 °C, indicating a progressive loss of thermal stability with increasing temperature. In contrast, the SAPEAS separator demonstrated outstanding dimensional integrity and maintained structural coherence even at elevated temperatures up to 200 °C, which is attributed to the excellent high-temperature resistance of the PA with a continuous network structure. This excellent performance enables the SAPEAS separator to maintain a stable physical barrier even well above its shutdown temperature (105 °C), preventing internal short circuits due to shrinkage and effectively halting thermal runaway caused by extensive electrode contact and intense Joule heat. These results confirm that the functional coating effectively protects the pristine PE substrate from high-temperature degradation, substantially mitigating safety risks, particularly in high-power battery applications [38].

Figure 4c depicts the thermal decomposition curves of the two separators. As observed, both separators exhibit similar onset temperatures for mass loss. Nevertheless, the PE separator has completely decomposed at around 500 °C, whereas the SAPEAS separator still retains 53% of its mass at 600 °C. This relatively high thermal char yield is primarily ascribed to its inorganic Al_2_O_3_ component. Additionally, thermomechanical analysis (TMA) revealed a coefficient of thermal expansion (*α*) of 871.9 ppm·K^−1^ for the PE separator between room temperature and 100 °C, accompanied by significant dimensional deformation (Figure 4d). In comparison, the SAPEAS separator showed a substantially lower *α* value of 175.9 ppm·K^−1^ over a broader temperature range from 25 °C to 200 °C. Taken together, these results demonstrate that the enhanced thermal and thermomechanical stability of the SAPEAS separator relative to the PE. This characteristic effectively alleviates the safety concerns related to the high-temperature operation of lithium-ion batteries, thereby broadening their application scope, particularly in environments subject to thermal challenges.

The above analyses demonstrate that the SAPEAS separator provides a hierarchical safety mechanism to address thermal abuse. The primary defense mechanism is an active shutdown at approximately 105 °C, where the melting of PEWMs blocks the ion-transport pathways to suppress electrochemical heat generation. In case the temperature continues to rise because of external factors, the secondary defense mechanism, passive containment, becomes critical. The excellent dimensional stability prevents separator collapse and massive internal short circuits, which are typical features of thermal runaway propagation. This combination of early intervention and robust high-temperature integrity offers a more comprehensive thermal safety solution than conventional separators.

An ideal separator should possess sufficient mechanical robustness to withstand high tension during battery assembly (in both cylindrical and pouch cell configurations) and accidental impacts, while effectively preventing internal short circuits caused by debris from rough electrode surfaces or lithium dendrite growth [41]. As shown in Figure 4e, the SAPEAS separator exhibits a tensile strength of 212.3 MPa, representing a 46% improvement compared to the PE separator (145.4 MPa), with an elongation at break maintained at 53.7%. Furthermore, the puncture strength of the SAPEAS separator reaches 0.64 kgf (Figure 4f), significantly exceeding that of the PE separator (0.54 kgf). This mechanical superiority arises from the synergistic effect between the rigid Al_2_O_3_-modified layer and the robust aramid continuous network structure, which together enhance the structural integrity of the PE separator under mechanical stress.

This enhanced structural integrity improves the resistance to mechanical damage via two mechanisms. Firstly, a preventive mechanism exists where the higher puncture strength directly elevates the breakdown threshold of the separator. Secondly, a mitigation mechanism is present in which the layered composite structure can not only suppress crack propagation to restrict the short circuit area but also augment the short circuit impedance after damage, thus alleviating the consequences of short circuits. Collectively, these characteristics enable the SAPEAS separator to endure mechanical stresses (e.g., battery expansion) and withstand electrochemical threats (e.g., lithium dendrite growth) during long-term cycling, thereby improving lithium-ion battery performance (cycle life) and safety (short-circuit prevention) [36,42].

### 3.4. Electrolyte Wettability, Electrochemical Stability, and Ion Transport Properties

**Figure 5:**
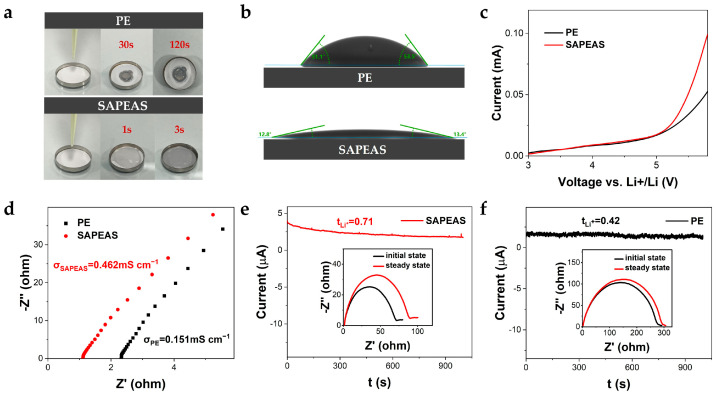
Electrochemical performance evaluation of SAPEAS separator. (**a**) Photograph and (**b**) Contact angle of PE and SAPEAS separators with liquid electrolyte. (**c**) LSV curves of the Li|separator|SS cells with PE and SAPEAS separators. (**d**) Nyquist plots of the SS|separator|SS symmetric cells with PE and SAPEAS separators. (**e**) Chronoamperometry curve and the corresponding EIS spectra (inset: before and after polarization) for the Li|separator|Li cell with SAPEAS separator. (**f**) Corresponding chronoamperometry curve and EIS spectra for the cell with PE separator.

Separators with excellent wettability are crucial for enabling batteries to achieve low internal resistance, high power output, long cycle life, and improved safety [43]. As shown in Figure 5a, the liquid electrolyte exhibits minimal penetration on the surface of the PE separator, in contrast to its complete spreading over the SAPEAS separator. Figure 5b further reveals that the contact angle of the PE separator remains at 53.5° after 60 s, whereas that of the SAPEAS separator rapidly decreases to 12.8° within just 3 s, indicating significantly enhanced wettability. The improved wettability of the SAPEAS separator toward liquid electrolytes can be attributed to the tightly interconnected porous structure of the PA layer, as well as the favorable compatibility between the hydroxyl and amide groups and the polar liquid electrolyte [44].

Electrochemical stability is a fundamental requirement for separators in lithium-ion batteries [41], which can be evaluated using linear sweep voltammetry (LSV) [45]. As shown in Figure 5c, the LSV curves of both the PE and SAPEAS separators exhibit substantial overlap up to a potential of around 5 V versus Li^+^/Li. This overlap indicates that these two separators possess comparable anodic stability within this voltage range, which fully encompasses the operational window of high-voltage cathodes. Beyond 5 V, the SAPEAS separator exhibits a sharper current rise, likely due to the electrochemical activity of the functional coating materials at extreme potentials well above typical battery operating ranges. These results imply that the SAPEAS separator can still maintain sufficient electrochemical stability within the typical operating voltage window of practical lithium-ion battery systems.

Ionic conductivity (*σ*) and lithium ion transference number (*t*_Li_^+^) are two key performance indicators for evaluating the ion transport efficiency of battery separators [36]. Electrochemical impedance spectroscopy (EIS) measurements revealed that the bulk resistances of the PE and SAPEAS separators were 1.1 Ω and 2.33 Ω, respectively (Figure 5d). The *σ* of the SAPEAS separator was calculated to be 0.462 mS cm^−1^, significantly higher than that of the PE separator (0.151 mS cm^−1^). Furthermore, the *t*_Li_^+^ was determined using Li|Li symmetric cells through a combined method of AC impedance and chronoamperometry (CA) with a small direct-current (DC) polarization. As depicted in Figure 5e,f, the CA curves illustrate the evolution of current during polarization. When a DC bias is applied, the current rapidly decreases and then stabilizes at a steady-state value. The corresponding EIS spectra (shown in the insets) before and after polarization reveal the alteration in interfacial resistance. Based on the initial current (*I*_0_), the steady-state current (*I*_s_), and the interfacial resistances (*R*_0_ and *R_s_*) obtained from these measurements, the lithium-ion transference number (*t*_Li_^+^) was calculated using Equation (2). The SAPEAS separator exhibited a superior *t*_Li_^+^ of 0.71, markedly higher than the 0.42 observed for the PE separator. Based on these results, the Li^+^ conductivity (*σ*_Li_^+^ = *σ* × *t*_Li_^+^) of the PE and SAPEAS separators was calculated to be 0.063 mS cm^−1^ and 0.328 mS cm^−1^, respectively.

Under normal conditions, lithium ions in the electrolyte are solvated by highly polar organic solvents (e.g., ethylene carbonate, EC), forming an extensive solvation sheath [46]. This reduces the concentration of free Li^+^ and hinders their trans-separator migration, potentially resulting in inefficient PF_6_^−^ anion transport and other issues such as concentration polarization, lithium dendrite growth, and thermal runaway [47]. Notably, the polar hydroxyl groups (-OH) originating from Al_2_O_3_, together with the amide groups (-CONH-) contributed by PA, synergistically strengthen ion-solvent interactions to construct a selective Li^+^ conduction environment [38], while its high mechanical strength of 212.3 MPa effectively suppresses lithium dendrite penetration and maintains structural integrity during cycling. These synergistic characteristics collectively reduce the occurrence of micro-short circuits even under high current densities, indirectly optimizing Li^+^ migration efficiency.

**Figure A1:**
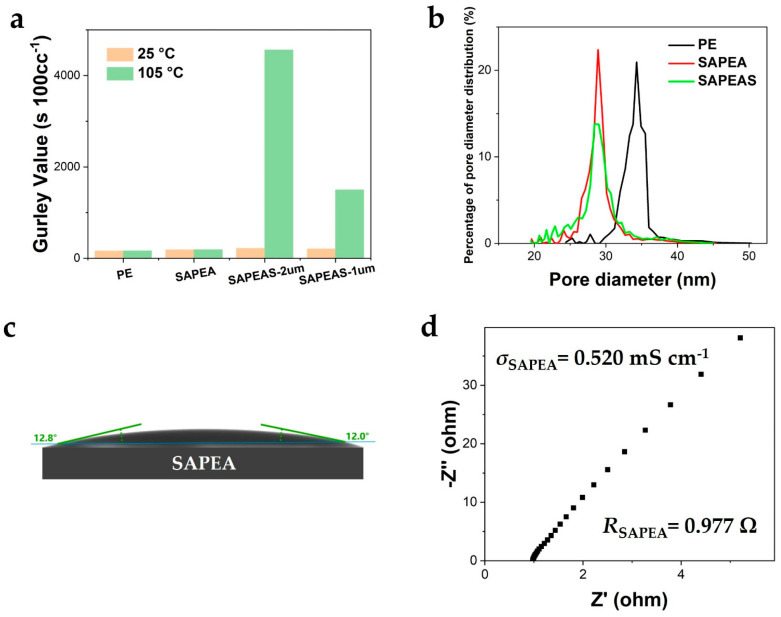
Analysis of the influence of coating thickness and the function of the PEWM layer: (**a**) Gurley value of separators; (**b**) The pore size distribution curves of separators. (**c**) Contact angle of SAPEA separator with liquid electrolyte. (**d**) Nyquist plots of the SS|separator|SS symmetric cells with SAPEA separator.

To guarantee the feasibility of the SAPEAS multi-layer separator in practical applications, we concentrated on examining the influence of the thickness of the polyethylene wax microspheres (PEWMs) layer, which directly dictates the thermal shutdown efficiency. By controlling the single-layer thickness of each functional layer within 1–3 μm (similar to the range of commercial multi-layer separators), separators with PEWM layer thicknesses of 1 μm (SAPEAS-1μm) and 2 μm (SAPEAS-2μm) were fabricated and compared with the control sample lacking the PEWM layer (SAPEA) and the original PE separator. As depicted in Figure A1a, at high temperatures, the Gurley value of the SAPEAS-2μm separator rose to 4560 s 100cc^−1^, which was substantially higher than that of SAPEAS-1μm (1498 s 100cc^−1^), suggesting that it possesses a more effective pore-blocking ability, thereby ensuring a more dependable thermal shutdown response. Meanwhile, the Gurley value (218.1 s 100cc^−1^) and average pore size (29 nm) of SAPEAS-2μm at room temperature (Figure A1b) still stayed within a reasonable range appropriate for battery applications and did not differ significantly from those of the SAPEA separator (Gurley value: 186.5 s 100cc^−1^, average pore size: 29 nm). Hence, 2 μm was identified as the optimal thickness of the PEWM layer, which minimizes the negative impact on ion transport kinetics to the greatest extent while ensuring a robust safety function.

To evaluate the potential impact of the PEWM coating on electrical performance, we characterized the separator without the PEWM layer (designated as SAPEA). As depicted in Figure A1c,d and Figure 5b, the SAPEA separator demonstrates excellent liquid affinity (contact angle: 12.8°) and high ionic conductivity (0.52 mS cm^−1^), which is comparable to that of the SAPEAS separator. This clearly verifies that the improvement of the basic electrochemical performance primarily originates from the hydrophilic alumina/aramid composite layer. In contrast, the ionic conductivity of the SAPEAS separator experiences a slight decline. This is a minor and justifiable cost for introducing the PEWM layer to attain the crucial thermal shutdown function. This control experiment clearly indicates that the PEWM layer is the exclusive source of the active safety mechanism, and its influence on the basic ion transport performance is manageable.

### 3.5. Battery Performances and Safety

**Figure 6:**
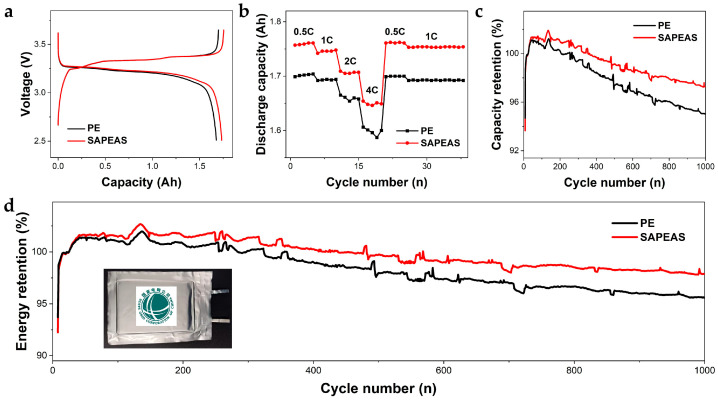
Electrochemical performance of LiFePO_4_/separator/Gr pouch full cells at room temperature. (**a**) Charge and discharge curves, (**b**) rate capability, and (**c**,**d**) cycling stability of cells using SAPEAS and PE separators.

To evaluate the practical application performance, LiFePO_4_|Gr pouch cells were assembled using SAPEAS and PE separators, respectively, and their electrochemical and safety performance was systematically assessed. As shown in Figure 6a, the cell with the SAPEAS separator delivers an initial discharge capacity of 1.76 Ah, higher than that of the PE-based cell (1.70 Ah). This improvement can be attributed to the greater liquid electrolyte uptake, which facilitates more thorough wetting of the electrode materials and benefits the lithium-ion intercalation/deintercalation processes, thereby yielding higher cathode utilization and reversible capacity [48].

Superior rate capability is essential for batteries operating under demanding scenarios such as fast charging and high-power discharge. Rate performance analysis reveals that the SAPEAS-based cell achieves specific capacities of 1.761, 1.748, 1.707, and 1.651 Ah at current rates ranging from 0.5C to 4.0C, respectively (Figure 6b). Notably, upon returning to 1C, the capacity fully recovers. The cell with the SAPEAS separator consistently outperforms the PE-based counterpart across all discharge rates and test conditions, which can be ascribed to its superior ion transport capability and enhanced electrolyte wettability, effectively reducing cell polarization even under high discharge rates [49].

The cycling stability of cells with different separators was also evaluated. The SAPEAS-based pouch cell retains 97.3% of its initial capacity and 97.9% of its energy after 1000 cycles at 0.5C (Figure 6c,d), whereas the PE-based pouch cell retains 95.0% and 95.6% of capacity and energy, respectively. These results indicate that the SAPEAS separator significantly enhances the cycling performance of lithium-ion batteries. This outstanding long-term cyclability can be attributed to the synergistic effects of multiple intrinsic characteristics. These include a uniform lithium-ion flux and low interfacial impedance resulting from rapid and stable electrolyte wettability, effective prevention of dendrite penetration and separator deformation due to the enhanced mechanical strength, and reduction in cycling polarization promoted by higher lithium-ion transference capability. Considering that Al_2_O_3_, PA, and PEWMs are all commercially available materials and that the SAPEAS separator is fabricated through well-established industrial processes, a production yield of approximately 88% has been achieved at the laboratory scale. With systematic engineering optimization for industrial-scale manufacturing (e.g., slurry batch consistency, proper web handling, and effective inline inspection), the yield is projected to reach and potentially exceed 88%, underscoring its promising potential for commercial application.

**Figure 7:**
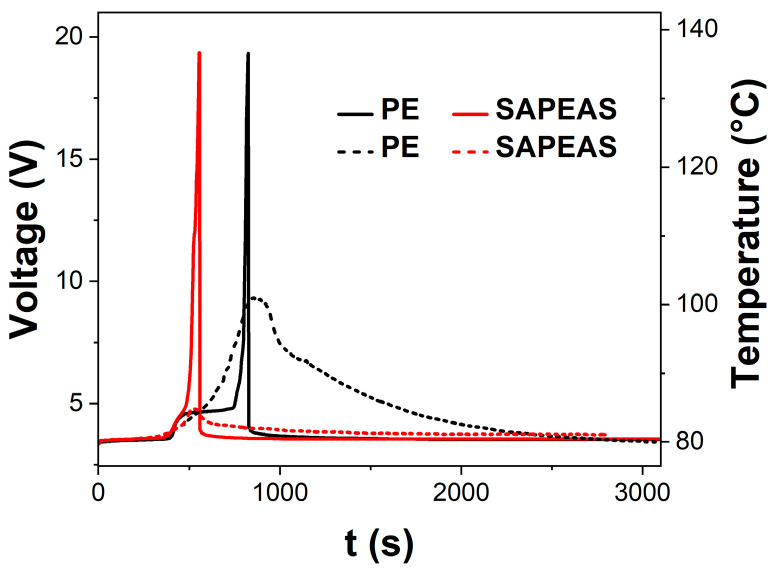
Comparison of overcharge performance of the pouch cells based on PE and SAPEAS. Temperature and voltage curves of the full cells during the overcharge test.

**Figure A2:**
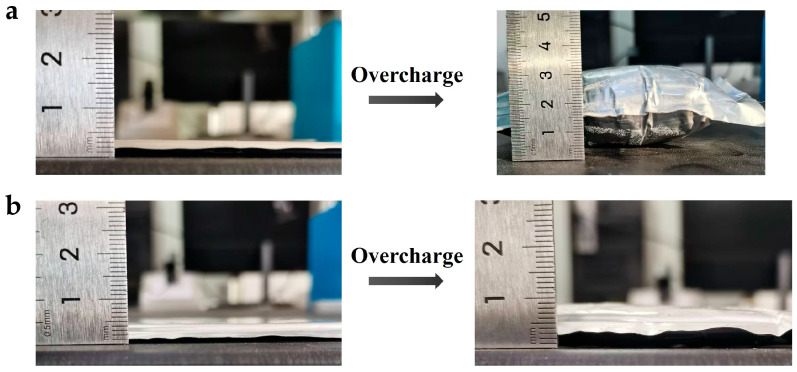
Photographs of the cell thickness measurements before and after overcharging: (**a**) PE-based cell; (**b**) SAPEAS-based cell.

**Figure A3:**
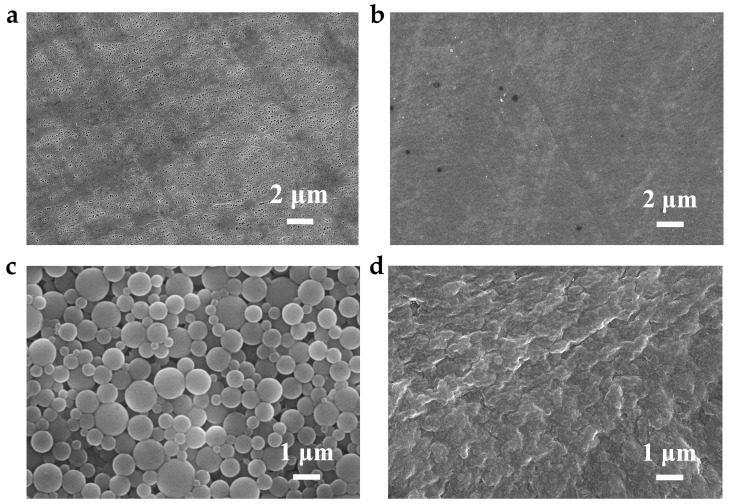
The SEM images of separators: (**a**,**b**) PE-based cell; (**a**) before overcharge test; (**b**) after overcharge test; (**c**,**d**) SAPEAS-based cell; (**c**) before overcharge test, (**d**) after overcharge test.

Overcharging is a common failure mode in LIBs and is closely related to the shutdown function of the separator, highlighting the positive role of the SAPEAS separator in improving battery safety. Figure 7 presents the temperature and voltage curves during overcharge testing. Initially, the curves for both cells overlap closely within the first 6 min. Subsequently, the voltage of the SAPEAS-based battery surges sharply and hits the cutoff voltage (19.2 V) at 9.3 min. Meanwhile, the corresponding surface temperature rises at a considerably lower rate, reaching a final peak of merely 85 °C. In sharp contrast, the voltage of the PE-based battery keeps rising until the cutoff is activated at 13.8 min, and its surface temperature ascends rapidly to 101 °C. It should be noted that the discrepancy between internal and external temperatures arises from poor heat dissipation, resulting in an internal temperature substantially higher than the monitored surface temperature [30]. After overcharging, the PE-based cell exhibits obvious bulging with the thickness expansion of approximately 843% (Figure A2a), whereas the SAPEAS-based cell shows slight expansion with the thickness expansion of approximately 157.1% (Figure A2b). SEM images in Figure A3a,b show that there are no visible pores left on the surface of the PE separator after overcharging, while the thermal-sensitive microspheres on the SAPEAS separator surface have melted (Figure A3c,d), indicating that pore closure has occurred in both separator types, which is consistent with the observed rapid voltage rise [50].

The earlier voltage cutoff, lower peak temperature, and significantly reduced swelling in SAPEAS-based batteries can be ascribed to the earlier activation of an irreversible active pore-blocking mechanism [51]. When the internal temperature of the battery rises during overcharging and reaches the melting point of PEWMs, these microspheres melt and spread, irreversibly obstructing the ion transport channels in the separator. This results in an increase in battery impedance, rapidly halting electrochemical reactions and thus suppressing subsequent intense exothermic and gas-generation processes. The synergistic combination of the active rapid-shutdown function and passive dimensional stability at high temperatures forms a hierarchical safety architecture of the SAPEAS separator, ultimately considerably enhancing the battery’s overcharge tolerance. By the end of the overcharge test, the cell assembled with SAPEAS exhibited 22% less electricity than PE, indicating enhanced safety during overcharging. These results demonstrate that the SAPEAS separator significantly enhances the overcharge safety of lithium-ion batteries, showing promising potential for the development of safer battery systems.

## 4. Conclusions

In summary, this work presents a scalable multilayer separator named SAPEAS, developed by sequentially constructing functional coatings of Al_2_O_3_, PA, and PEWMs on a PE substrate, which effectively integrates early thermal shutdown capability, high-temperature dimensional stability, enhanced mechanical strength, and improved electrochemical performance. In actual pouch-type LFP|Gr cells with ampere-hour (Ah) level capacity, owing to the above-mentioned comprehensive advantages, this separator exhibits excellent cycling stability and safety performance, thereby clearly demonstrating its potential for high-safety, high-performance LIBs.

Beyond the remarkable laboratory-scale performance, the SAPEAS separator shows obvious potential for industrial application, primarily due to its scalable fabrication method. The key manufacturing step—microgravure coating—is a well-established, high-speed, roll-to-roll process commonly used in precision coating industries. This enables a seamless transition from lab-scale prototypes to continuous, meter-wide production, offering a distinct advantage over many complex or low-throughput nanofabrication approaches. Moreover, the process uses commercially available materials (such as Al_2_O_3_) and does not require extreme processing conditions.

Instead of replacing conventional polyolefin separators, SAPEAS enhances their functionality through an industrially compatible coating process. This “drop-in” modification aligns with existing battery manufacturing infrastructure, thereby minimizing the need for capital-intensive retooling. The probability of adoption is anticipated to be highest in market segments where enhanced safety and superior electrochemical performance are prioritized.

Future work will focus on optimizing the coating formulation for higher speeds and assessing scalability through detailed techno-economic analysis. Such scalable, multifunctional designs are expected to advance the safety and electrochemical performance of next-generation LIBs.

## Data Availability

The original contributions presented in this study are included in the article. Further inquiries can be directed to the corresponding author.
